# Driving Oscillatory Activity in the Human Cortex Enhances Motor Performance

**DOI:** 10.1016/j.cub.2012.01.024

**Published:** 2012-03-06

**Authors:** Raed A. Joundi, Ned Jenkinson, John-Stuart Brittain, Tipu Z. Aziz, Peter Brown

**Affiliations:** 1 Functional Neurosurgery and Experimental Neurology Group, Nuffield Department of Clinical Neurosciences, University of Oxford, Oxford OX3 9DU, UK; 2 Department of Physiology, Anatomy and Genetics, University of Oxford, Oxford OX1 3QX, UK; 3 Centre of Excellence in Personalised Healthcare, Institute of Biomedical Engineering, Department of Engineering Sciences, University of Oxford, Oxford OX3 7DQ, UK; 4 Nuffield Department of Surgery, John Radcliffe Hospital, Oxford OX3 9DU, UK

## Abstract

Voluntary movement is accompanied by changes in the degree to which neurons in the brain synchronize their activity within discrete frequency ranges. Two patterns of movement-related oscillatory activity stand out in human cortical motor areas. Activity in the beta frequency (15–30 Hz) band is prominent during tonic contractions but is attenuated prior to and during voluntary movement [1]. Without such attenuation, movement may be slowed, leading to the suggestion that beta activity promotes postural and tonic contraction, possibly at a cost to the generation of new movements [2, 3]. In contrast, activity in the gamma (60–90 Hz) band increases during movement [4]. The direction of change suggests that gamma activity might facilitate motor processing. In correspondence with this, increased frontal gamma activity is related with reduced reaction times [5]. Yet the possibility remains that these functional correlations reflect an epiphenomenal rather than causal relationship. Here we provide strong evidence that oscillatory activities at the cortical level are mechanistically involved in determining motor behavior and can even improve performance. By driving cortical oscillations using noninvasive electrical stimulation, we show opposing effects at beta and gamma frequencies and interactions with motor task that reveal the potential quantitative importance of oscillations in motor behavior.

## Highlights

► Cortical driving at 20 and 70 Hz slows and speeds voluntary force generation ► Performance can be enhanced even during contractions made as fast as possible ► Scale of effects of cortical driving depend on the nature of the cued motor task

## Results

**Figure 1:**
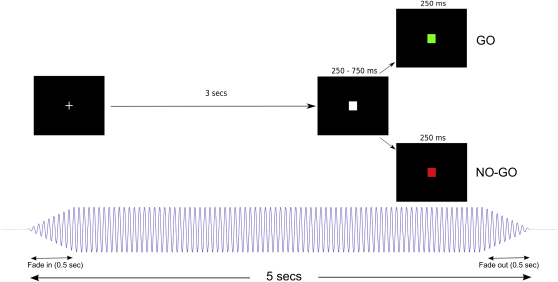
Schematic of Paradigm Used for Go/No-Go Task A fixation cross is presented that triggers the onset of sinusoidal stimulation shown below. After 3 s, a square white precue was presented followed quickly (250–750 ms, randomized) by either a square green go cue or red no-go cue, which lasted 250 ms. Subjects were instructed to squeeze as quickly and as hard as they could in response to the go cue and withhold their response on no-go cues. Stimulation had a 0.5 s ramp up and down and lasted for a total of 5 s, which meant that it continued throughout the behavioral response and faded away shortly thereafter. There was then a 6 s delay between the response cue and subsequent fixation cross for the next trial, during which the subject was at rest.

To demonstrate that oscillatory cortical activities can modify motor behavior, we used transcranial alternating current stimulation (TACS) to entrain oscillatory activity in the cortex with low amplitude currents at specified frequencies in a completely noninvasive manner [3]. Such stimulation has been demonstrated to increase oscillatory activity in the brain at the frequency of stimulation [6]. Thus, we were able to investigate how such stimulation modulated motor performance in a go/no-go paradigm, which contrasts two very different cue-determined tasks, action and motor inhibition. Eighteen healthy subjects who were unable to detect periods of active stimulation were asked to hold a grip force sensor (MIE Medical Research, UK) with their right hand while seated and attending to a go/no-go task presented on a computer screen in front of them (Figure 1). A precue was followed immediately by either a green square (go signal) or a red square (no-go signal), randomly selected with a 2:1 ratio of go to no-go cues. Subjects were instructed to squeeze the force sensor as hard and fast as they could in response to the go cue and refrain from responding to the no-go cue. Stimulation was randomly applied in 50% of trials.

### Stimulation at 20 and 70 Hz Has Opposing Effects in Go Trials

**Figure 2:**
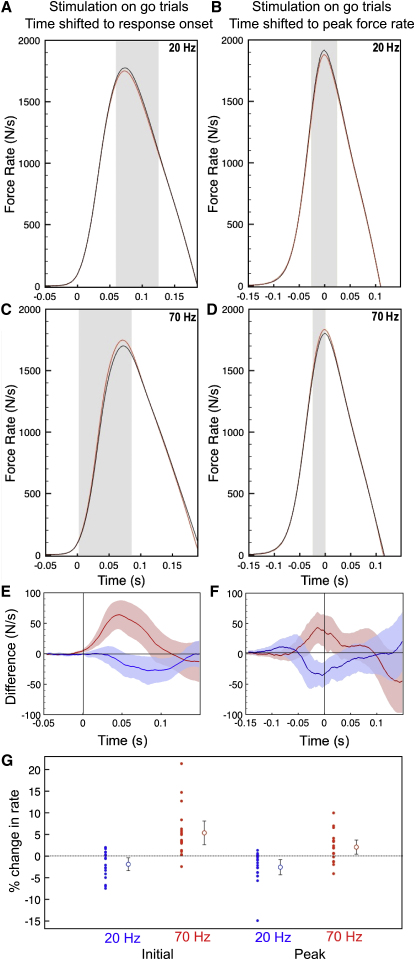
Effect of Stimulation on Go Trials (A–D) In (A) and (B), stimulation is applied at 20 Hz and in (C) and (D) at 70 Hz. Black traces show no-stimulation trials and red traces stimulation trials. Traces are grand averages of all subjects' force rates aligned to the point of first development of 5% peak force rate (A and C) or peak rate of force generation (B and D). Vertical gray bars demonstrate areas of significant difference between stimulation and no stimulation (serial two-tailed paired t tests, p < 0.05). (E–G) The mean differences with confidence intervals of ±2 SEM between stimulation and no-stimulation conditions for 20 Hz (blue) and 70 Hz (red), aligned as above. Vertical lines at time 0 represent the point of first development of 5% peak force rate (E) and point of peak force rate (F). In (G), percent changes for each subject are shown for both initial rate and peak rate, for 20 Hz (blue) and 70 Hz (red) stimulation. Adjacent to individual changes are mean changes with ±2 SEM. See also Figure S1 for example of individual traces.

There was no change in reaction time or peak force during either 20 or 70 Hz stimulation (see Table S1 available online). However, stimulation at 20 Hz reduced the initial rate of force development by 1.89% ± 0.74% compared to no stimulation (t_[17]_ = −2.541, p = 0.0211, unpaired t test; drop from 1,036 ± 101 N/s [SEM unless stated otherwise] to 1,024 ± 103 N/s, t_[17]_ = 1.781, p = 0.1057; Figures 2A, 2E, and 2G) and led to a reduction in peak force rate of 2.56% ± 0.87% (t_[17]_ = −2.934, p = 0.0092; drop from 1,919 ± 176 N/s to 1,883 ± 179 N/s, t_[17]_ = 3.414, p = 0.0033; Figures 2B, 2F, and 2G).

Gamma stimulation had the opposite effect. The initial rate of force development increased by 5.37% ± 1.3% (t_[17]_ = 3.92, p = 0.0011; rise from 996 ± 105 N/s to 1,043 ± 109 N/s, t_[17]_ = −4.95, p = 0.0001; Figures 2C, 2E, and 2G). Peak force rate increased by 2.08% ± 0.82% relative to no stimulation (t_[17]_ = 2.537, p = 0.0212; rise from 1,806 ± 171 N/s to 1,837 ± 170 N/s, t_[17]_ = −2.32, p = 0.0344; Figures 2D, 2F, and 2G).

### Effects of 20 Hz Stimulation Were Dramatically Increased in No-Go Trials

Our paradigm elicited contrasting intentions: action on go trials or motor inhibition on no-go trials. During no-go trials, false responses provided a behavioral measure of performance in which motor inhibition was only partially successful. Such errors of commission were absent in four out of the 18 subjects and in the remaining subjects occurred in an average of 45% of trials (Table S2). In these subjects, peak force in error trials was far smaller than in go trials, confirming that motor inhibition was present, but not entirely successful during these trials (40.0 ± 9.9 N versus 212.0 ± 11.7 N, p < 0.001, paired t test).

**Figure 3:**
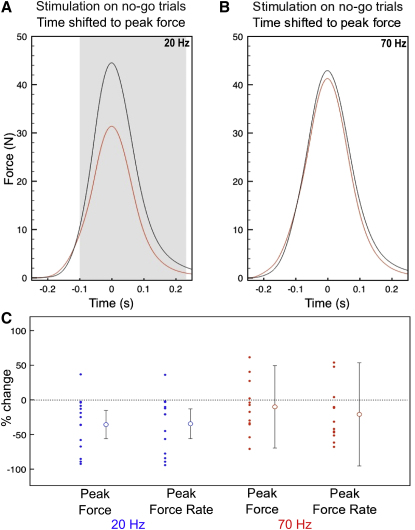
Effect of Stimulation on No-Go Trials Grand averages for no-go trials aligned to peak force are displayed for 20 Hz (A) and 70 Hz (B), with the gray bar showing an extended period of significant suppression for 20 Hz but not 70 Hz. Individual percent changes are shown in (C) for 20 Hz (blue) and 70 Hz (red) for peak force and peak rate of force generation along with means ±2 SEM (displayed with two outliers not shown from gamma peak force, and one from gamma peak velocity. Outliers are shown in Figure S1). See also Figure S1 for example of individual traces.

Beta stimulation led to a dramatic 34.5% ± 10.7% drop in the peak rate of force development in error trials relative to no stimulation (t_[13]_ = −3.215, p = 0.0068; from 607 ± 135 N/s to 413 ± 127 N/s, t_[13]_ = 4.31, p = 0.00084; Figure 3C). A comparable 35.7% ± 10.2% decrease in peak force was seen (t_[13]_ = −3.49, p = 0.004; from 462 ± 109 N to 316 ± 104 N, t_[13]_ = −4.45, p = 0.00053; Figures 3A and 3C). The effects of gamma stimulation were more variable and showed no significant difference in either peak force (32.2% ± 31.2%, t_[13]_ = 1.033, p = 0.3204), or peak rate of force generation (14.8% ± 37.2%, t_[13]_ = 0.3891, p = 0.6971; Figure S1). This variability was mainly due to three results from two subjects who had percent increases more than four times the standard error from the mean during gamma stimulation. However, the effect of gamma stimulation remained insignificant even when these results were eliminated (Figure 3C; Table S3).

### Despite Different Effect Sizes, Responses to 20 Hz Stimulation Are Correlated in Go and No-Go Trials

**Figure 4:**
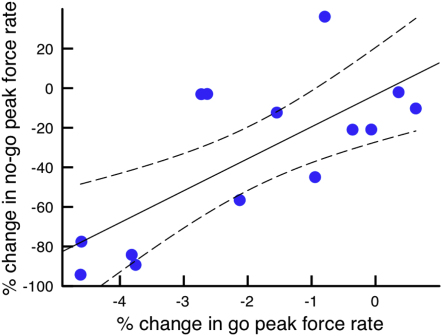
Correlation and 95% Confidence Intervals between Go and No-Go Trials with 20 Hz Stimulation Percent change in peak force rate is significantly correlated between go and no-go trials, suggesting a common inhibitory effect of 20 Hz stimulation (r = 0.728, p = 0.0032; n = 14 as errors of commission were absent in no-go trials in four of the 18 subjects).

The impairment of force generation in both go and erroneous no-go trials with 20 Hz stimulation raised the question: are these effects related? In line with this, there was a significant correlation between percent change of peak rate of force generation in go trials and no-go trials during 20 Hz stimulation, (r = 0.728, p = 0.0032, Figure 4) with no such correlation for 70 Hz (r = 0.270, p = 0.35), suggesting a relationship between the mechanisms of force rate inhibition.

## Discussion

There is considerable evidence that oscillatory activity in the brain is modulated in a task-specific manner [7–10]. However, whether synchronized oscillations arise as an epiphenomenal product of brain physiology, or are causal to our behavior, remains an open question. The frequency-dependent bidirectional influence of cortical entrainment on motor control shown here, and its quantitative dependence on the motor task triggered by the imperative cue, lend strong support to the possibility that, at least in the motor domain, synchronized oscillations are fundamental to brain function.

We have previously demonstrated that TACS at similar intensities does drive motor cortical oscillations as evidenced by changes in corticomuscular coherence [3], but could it be that changes in cortical excitability also occur and actually underscore the behavioral effects? This seems unlikely because the tonic grip force before the warning cue remained unchanged during stimulation. In addition, TACS at 80 Hz has no impact on cortical excitability after as long as 10 min of stimulation at 1,000 μA [11]. TACS at 20 Hz can, following 90 s of stimulation, selectively increase motor cortical excitability at rest [12], which is difficult to reconcile with the reduction in the rate of force generation shown here. Perhaps the effects on behavior are independent and the excitability changes require longer periods of stimulation. Alternatively, and as suggested by the authors of the excitability study, entrainment-induced excitability of a population of neurons at beta frequency may come at the expense of selective motoneuronal recruitment, thereby impairing performance [12]. Could stimulation have brought nonspecific attentional processes to bear? We were careful to keep stimulation below perceptual threshold and, in line with this, reaction time was unaffected by stimulation. Moreover, any shift in attention due to subliminal scalp sensation or phosphenes could not readily explain the opposite effects of stimulation at different frequencies.

We elected to entrain motor cortical function at two specific frequencies so as to test the hypothesized contrasting roles of oscillatory activities in the “antikinetic” beta and “prokinetic” gamma bands. We chose these particular frequencies because oscillatory activity in the motor cortex during movement is commonly centered around 20 Hz [7] and 70 Hz [4]. Beta activity in the motor system has been considered to be antikinetic in so far as it is associated with slower voluntary movements both in health [2] and disease [13]. Conversely, gamma activity has been suggested to be prokinetic given that it is increased in the basal ganglia-cortical motor loop during voluntary movement [14]. Our experimental manipulations support this dissociation, albeit a convenient but gross simplification of the behavioral relevance of oscillations in the corticobasal ganglia system. In particular, it must be stressed that both the beta and, especially, the gamma band are very wide and have the potential to encompass oscillatory activities with different functional roles, according to their precise spatiospectral characteristics [8].

One of the remarkable observations made here is the interaction between cortical entrainment at 20 Hz and the motor task triggered by the imperative cue. Stimulation at 20 Hz afforded a significant but modest slowing of force production in the go task, akin to the results of Pogosyan et al. [3]. However, stimulation in no-go trials, where the triggered motor task involved inhibition, led to a major reduction in force generation during errors of commission in a performance-enhancing direction. Nevertheless, the behavioral effects during go and no-go trials with 20 Hz stimulation were related as indicated by their correlation across subjects. Several studies have now suggested that sensorimotor cortical areas may have a natural resonance frequency of about 20 Hz [12, 15, 16]. The implication is that stimulation interacts with this rhythm to drive oscillations [12, 16, 17] but that the degree to which resonance phenomena are damped in the cortex is dynamically determined by task demands. In our paradigm, the latter are set by the imperative cue to either action or motor inhibition. Voluntary action was associated with a modest effect of 20 Hz stimulation and, as previously noted, is well established to be preceded and accompanied by an attenuation of spontaneous beta oscillations in line with increased damping of cortical resonance in the beta band. In contrast, motor inhibition was associated with a pronounced effect of 20 Hz stimulation and is known to be preceded and accompanied by an increase in spontaneous beta oscillations in no-go trials at cortical [18] and subcortical [19] levels.

The performance enhancement was also seen in go trials, but only during stimulation with 70 Hz TACS. Such stimulation, in contrast to 20 Hz TACS, improved the rate of force generation, particularly early in the grip, effectively raising the subjects' mean maximal voluntary output. Although TACS in the gamma band has been shown to influence sensory function [20, 21], we show for the first time that motor behavior can be improved by imposing synchronized oscillatory activity upon motor cortical regions. The improvement in the rate of force generation was significant but relatively modest in size, perhaps because of a ceiling effect whereby performance could not be improved much more. Nevertheless, in conjunction with the results of 20 Hz stimulation, the findings provide proof of principle for the anti/pro-kinetic model of beta and gamma oscillations. In addition, the results with 70 Hz stimulation provide further evidence for a dynamic change in cortical susceptibility to oscillatory driving according to motor task. Improvements in force generation were only seen during go trials, i.e., when action was intended and spontaneous gamma activity is known to increase [4]. In contrast, 70 Hz TACS was ineffective during errors of commission following no-go cues, presumably a consequence of the reconfiguration of cortical resonance properties during motor inhibition. The basal ganglia are one system likely to regulate cortical damping and resonance [14].

Our observations are important in providing interventional evidence that oscillatory activity of the brain is causally linked to aspects of motor behavior but also in suggesting more targeted approaches to interventional treatments in diseases dominated by insufficient or excessive movement. In particular, the prominent effect of 20 Hz TACS in promoting the inhibition of unintended movements in no-go trials raises the possibility that similar stimulation of the cortex may be effective in suppressing unwanted or excessive output of the motor system, such as tics or dyskinesias.

## Experimental Procedures

The Oxfordshire Research Ethics Committee B approved the study, and all subjects gave informed written consent. Nineteen healthy subjects were recruited. The paradigm consisted of the presentation of a fixation cross which lasted 3 s, followed by a precue (Figure 1). The precue was displayed for between 250 and 750 ms and was followed immediately by either a go or a no-go signal lasting 250 ms, randomly selected with a 2:1 ratio. This ratio was chosen so as to increase the possibility of errors on no-go trials due to a heightened expectation of go cues. Sessions consisted of four blocks, each with 42 trials. Subjects were reminded before each block to remain vigilant and respond as quickly as possible to go cues. Stimulation was randomly applied in 50% of trials and ramped up over 0.5 s simultaneously with the presentation of the fixation cross. Stimulation was on for 3 s before the precue, and it lasted a total of 5 s before ramping down over 0.5 s. Stimulation was delivered using a bipolar current stimulator (DC-Stimulator Plus, NeuroConn, Ilmenau, Germany) via sponge electrodes soaked in saline. We placed the target electrode (area 5 × 7 cm) on the scalp overlying the hand area of the left motor cortex, as identified by single monophasic pulses of Transcranial Magnetic Stimulation (MagStim 200, Whitland, Wales, UK), and the reference electrode (5 × 10 cm) on the ipsilateral shoulder. Subjects came for two separate sessions in which either 20 or 70 Hz stimulation was delivered. Sessions were separated by approximately 7 days and the order of 20 and 70 Hz stimulation was counterbalanced.

### Analysis

For go trials, we determined response onset by thresholding force responses at 2% of maximum force output on a trial-by-trial basis. We aligned trials according to this response onset within subjects, and the thresholding latency was used to determine reaction time, with the exception that any responses made within 100 ms of target presentation were rejected. Any go-trials in which no response was made were also rejected. We then differentiated the force response to obtain rate of force, with trials where peak rate was outside 2 SD of the mean rejected. Collectively, these rejection criteria resulted in the exclusion of less than 10% of trials in any condition (Table S1). Moreover, there was no significant difference in the number of trials eliminated across conditions (Table S1). To determine the initial rate of force development, we realigned individual force rate averages across subjects according to 5% of peak force rate. To determine peak force and the peak rate of force development, we aligned force and differential force traces to peak values (see Figure S2 for example of individual averages during no stimulation). Grand averages were then constructed about both initial rate and peak rate.

## Supplemental Information

Document S1. Figures S1 and S2, Tables S1–S5, Supplemental Analysis, Supplemental Results, and Supplemental Experimental Procedures
