# Supplementary material for: Driving Oscillatory Activity in the Human Cortex Enhances Motor Performance
Source: Curr Biol. 2012 Mar 6;22(5):403–7. doi: 10.1016/j.cub.2012.01.024 (PMC3343257; doi:10.1016/j.cub.2012.01.024)
Supplement: Document S1. Figures S1 and S2, Tables S1–S5, Supplemental Analysis, Supplemental Results, and Supplemental Experimental Procedures [file mmc1.pdf]

Current Biology, Volume *22*

Supplemental Information

Driving Oscillatory Activity in the Human Cortex Enhances Motor Performance

Raed A. Joundi, Ned Jenkinson, John-Stuart Brittain, Tipu Z. Aziz, and Peter Brown

Supplemental Inventory

1. Supplemental Figures and Tables

Figure S1, related to Figure 3C

Figure S2, related to Figures 2A and 3A

Table S1, related to Results

Table S2, related to Results

Table S3, related to Results and Figure 3C

Table S4, related to Results

Table S5, related to Results

2. Supplemental Experimental Procedures

3. Supplemental Analysis

4. Supplemental Results

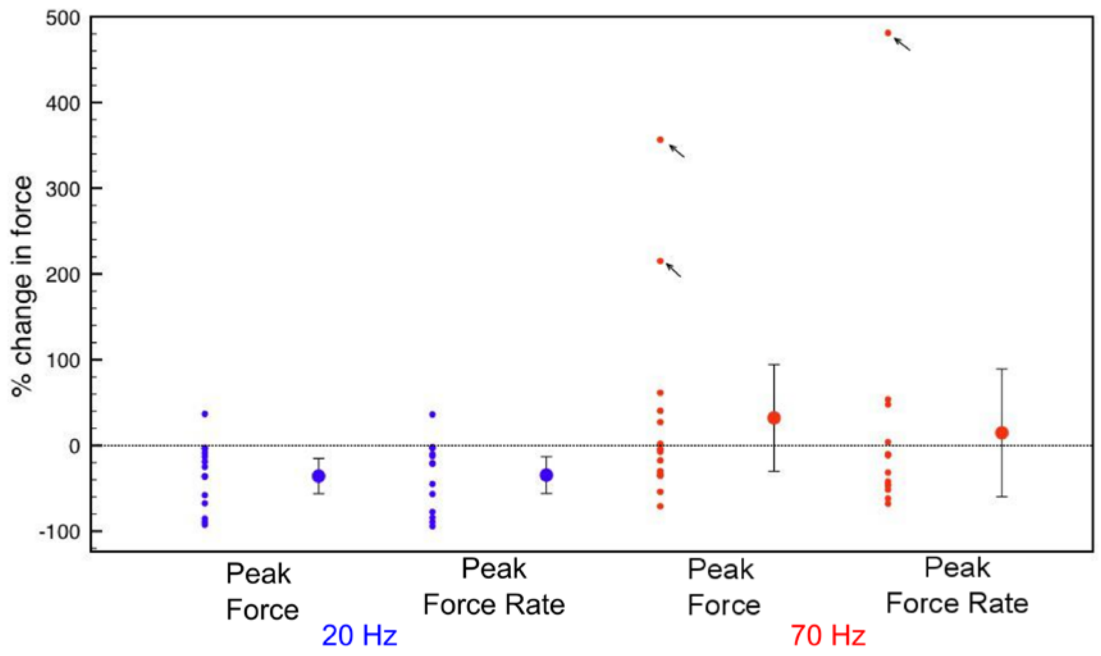

Figure S1. No-Go % Change with Outliers Included, Related to Figure 3C

Individual % changes are shown for 20 Hz (blue) and 70 Hz (red) for peak force and peak rate along with means $\pm$ 2 SEM. Outliers are identified with arrows.

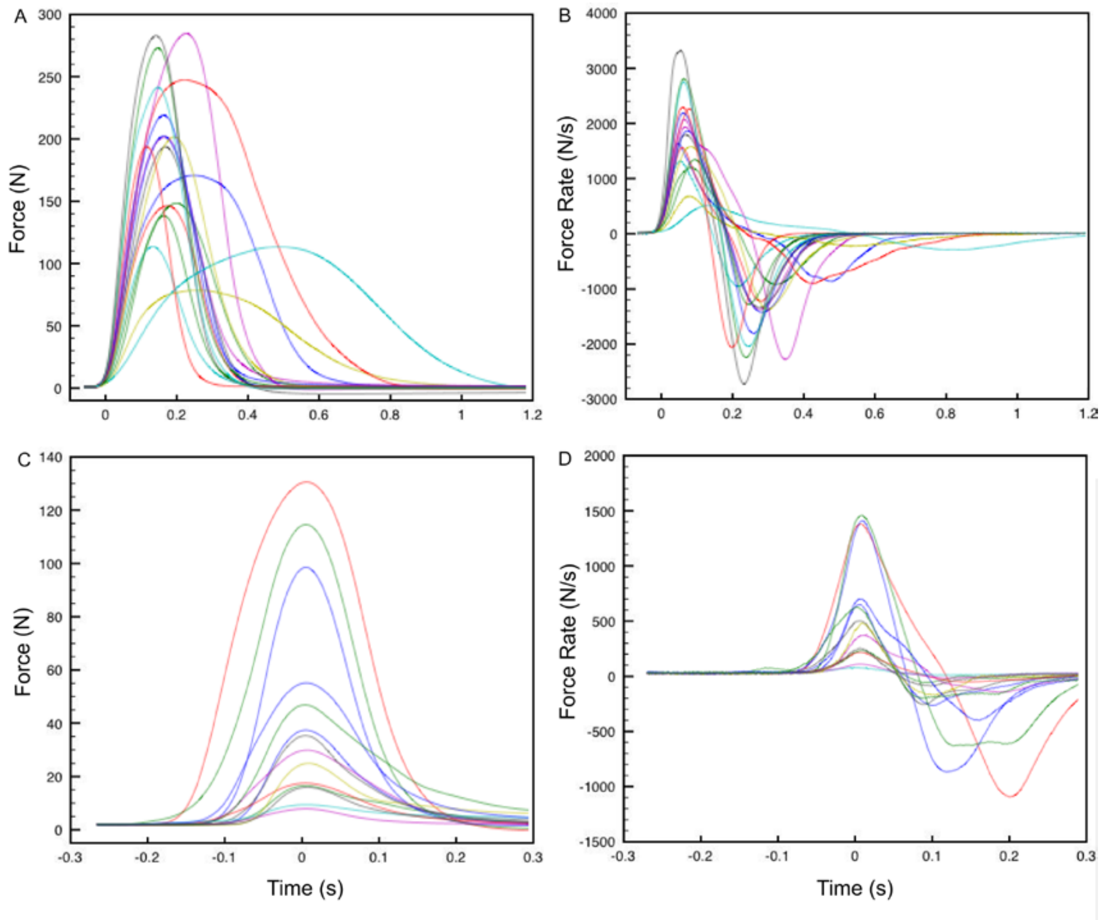

Figure S2. Individual Subject Averages during No-Stimulation, Related to Figure 2A and Figure 3A

Average traces per subject during no-stimulation trials in the 20 Hz session, shown for (A) go force aligned to onset, (B) go force rate aligned to onset, (C) no-go force aligned to peak, and (D) no-go force rate aligned to peak. Subjects are color matched across panels.

Table S1. Nonsignificant Differences for Go Trials, Related to Results

Condition	No Stimulation (mean ± SEM)	Stimulation	Significance (paired t-test)
**Go reaction time**			
20 Hz	271 ± 7.1 ms	268 ± 8.3 ms	P=0.373
70 Hz	266 ± 11 ms	262 ± 8.4 ms	P=0.416
**Go Peak force**			
20 Hz	200 ± 15 N	199 ± 15 N	P = 0.198
70 Hz	206 ± 17 N	207 ± 17 N	P = 0.546
**Percentage go trials rejected**			
20 Hz	6.5 ± 1.5	7.4 ± 1.7	P=0.511
70 Hz	5.8 ± 0.9	6.1 ± 1.0	P=0.705
**Force between fixation cross and pre-cue**			
20 Hz	9.3 ± 6.4 N	9.4 ± 6.4 N	P=0.319
70 Hz	10.0 ± 6.2 N	10.1 ± 6.2 N	P=0.655
**Force between pre-cue and response**			
20 Hz	9.3 ± 6.4 N	9.4 ± 6.4 N	P=0.311
70 Hz	10.0 ± 6.2 N	10.1 ± 6.2 N	P=0.580

Table S2. Percentage of Errors of Commission and Total Number of No-Go Trials per Stimulation Frequency, Related to Results

Condition	No Stimulation	Stimulation	Significance (paired t-test)
**Percentage relative to total number of trials**			
20 Hz	44.5% ± 6.1	41.6% ± 7.0	P=0.336
70 Hz	45.3% ± 5.3	45.0% ± 5.5	P=0.947
**Absolute number of trials**			
20 Hz	13.3 ± 1.7	12.8 ± 1.7	P=0.758
70 Hz	13.0 ± 1.4	12.6 ± 1.5	P=0.664

Table S3. Related to Results and Figure 3C

70 Hz no-go trials, outliers removed. Gamma stimulation remains insignificant.

**Parameter**	**% change**	**Significance**
Peak Force	-10.1 + 11.2	P=0.386
Peak Rate	-21.0 + 11.1	P=0.072

Table S4. Related to Results

Significant differences between 20 Hz and 70 Hz stimulation. Go and no-go trials were different for all parameters.

	Significance (paired t-test)
**GO**	
Initial velocity	P<0.001
Peak velocity	P<0.001
Time to peak	P=0.0037
**NO-GO**	
Peak force	P = 0.0193
Peak velocity	P = 0.0212

Table S5. Nonsignificant Correlations, Related to Results

There was no correlation between percent changes during 20 Hz and 70 Hz stimulation, or between intensity of stimulation and performance.

Parameter 1	Parameter 2	r value	Significance
**% change for 20 Hz and 70 Hz stimulation**			
Go trial initial rate with 20 Hz	Go trial initial rate with 70 Hz	-0.0356	P = 0.889
Go trial peak rate with 20 Hz	Go trial peak rate with 70 Hz	0.2359	P=0.346
**Intensity of stimulation and % change in performance**			
Intensity of Stimulation	Go initial rate with 20 Hz	0.0851	P=0.763
Intensity of Stimulation	Go peak rate with 20 Hz	0.0929	P=0.742
Intensity of Stimulation	Go initial rate with 70 Hz	-0.2125	P=0.447
Intensity of Stimulation	Go peak rate with 70 Hz	-0.1075	P=0.703
Intensity of Stimulation	Go peak force with 20 Hz	-0.2917	P=0.291
Intensity of Stimulation	Go peak force with 70 Hz	0.2987	P=0.280
Intensity of Stimulation	Nogo peak force with 20 Hz	0.1073	P=0.727
Intensity of Stimulation	Nogo peak rate with 20 Hz	0.0846	P=0.783
Intensity of Stimulation	No-go peak force with 70 Hz	-0.2589	P=0.442
Intensity of Stimulation	No-go peak rate with 70 Hz	-0.2405	P=0.476

Supplemental Experimental Procedures

During the first session, the subject's phosphene or scalp sensation threshold (whichever was lowest) to 20 Hz stimulation was determined, and an amplitude 50 µA below this was selected for subsequent testing stimulation. Impedance during all stimulation sessions was always kept below 15 mΩ. Sub-threshold stimulation was confirmed by a forced-choice task comprising of 20 rounds of stimulation. One subject was always aware when stimulation was being applied and was therefore excluded from the study. A further two subjects experienced scalp sensations or phosphenes when stimulated at the selected intensity in the second session and so the intensity in the second session was lowered by a further 100µA. There was no significant difference between the mean stimulus amplitude of the two sessions (903 ± 111 µA for beta and 915 ± 98 µA for gamma stimulation, $t_{[17]}$=-0.128, p=0.901, paired t-test).

To assess the subject's perception of the stimulation, we employed a force-choice test prior to the experiment in which the subjects would attend to the task cues but instead of making a motor response they would indicate whether or not they thought the stimulation was active each time a response cue was presented (either red or green). The stimulation setting (ON or OFF) and their response (ON or OFF) was recorded for 20 trials. We then coded correct responses as 1 and incorrect responses as 0 and performed a binomial test on each dataset. The p value was always insignificant and ranged from 0.2 to 1, with the exception of one case which was 0.077. This confirmed that subjects were not aware of any active stimulation during the task.

Supplemental Analysis

For no-go trials, we sought to analyze all trials in which some erroneous force response (ie 'twitch') had been produced. To do this we calculated the standard deviation of the baseline period, and rejected any trial in which the peak force was below 5 times this standard deviation. This allowed us to include all trials in which a small deviation from baseline occurred, but reject trials in which there was no response whatsoever. In most go/no-go experiments , the response is defined by an 'all-or-none' behavior, such as a button push, thus giving rise to a fairly low error rate, as compared with ours (mean 45%). Here, we were most interested in capturing the changes in force performance due to stimulation, and thus designated a low cutoff. We subsequently aligned trials to peak force and peak force rate, as in go trials.'

Supplemental Results

The percentage change with gamma stimulation was significantly greater with respect to the initial rate of force development than peak velocity ($t_{[17]}$ =3.39, p=0.0035), but not so for beta stimulation($t_{[17]}$=1.08, p=0.297).

Due to the faster development of force with gamma stimulation there was a 3.65± 0.86 % reduction in the time to achieve peak rate compared to no-stimulation ($t_{[17]}$ = -4.25, p =0.00053; drop from 81.3 ± 4.9 ms to 78.1 ± 4.3 ms , $t_{[17]}$ =3.69, p = 0.0018). No such change was apparent for beta stimulation (0.84 ± 0.63 %, $t_{[17]}$ = 1.327, p =0.202). There were significant differences in initial and peak rate when comparing beta and gamma stimulation, confirming a differential effect between the two interventions (Table S4). The independence of the effects of the two stimulation frequencies was suggested by the lack of any across-subject correlation in either initial or peak rate percentage change between beta and gamma stimulation (Table S5). In addition, there was no correlation between intensity of stimulation and any behavioral change (Table S5). Finally, there were no differences in grip force levels before the response cue was presented, regardless of the ongoing stimulation at 20 Hz or 70 Hz. The lack of modulation in baseline resting force suggests that stimulation per se did not simply alter the resting level of muscle activity (Table S1).
